# Infectious pancreatic necrosis virus triggers antiviral immune response in rainbow trout red blood cells, despite not being infective

**DOI:** 10.12688/f1000research.12994.2

**Published:** 2017-12-13

**Authors:** Ivan Nombela, Aurora Carrion, Sara Puente-Marin, Verónica Chico, Luis Mercado, Luis Perez, Julio Coll, Maria del Mar Ortega-Villaizan

**Affiliations:** 1 Instituto de Biología Molecular y Celular, Miguel Hernández University, Elche, Spain; 2 Institute of Biology, Catholic Pontifical University of Valparaiso, Valparaiso, Chile; 3 Instituto Nacional de Investigación y Tecnología Agraria y Alimentaria, Madrid, Spain

**Keywords:** erythrocytes, IPNV, birnavirus, immune response, antiviral, trout, interferon

## Abstract

**Background**: Some fish viruses, such as piscine orthoreovirus and infectious salmon anemia virus, target red blood cells (RBCs), replicate inside them and induce an immune response. However, the roles of RBCs in the context of infectious pancreatic necrosis virus (IPNV) infection  have not been studied yet.

**Methods**: Ex vivo rainbow trout RBCs were obtained from peripheral blood, Ficoll purified and exposed to IPNV in order to analyze infectivity and immune response using RT-qPCR, immune fluorescence imaging, flow cytometry and western-blotting techniques.

**Results**: IPNV could not infect RBCs; however, IPNV increased the expression of the INF1-related genes *ifn-1*, *pkr* and *mx* genes. Moreover, conditioned media from IPNV-exposed RBCs conferred protection against IPNV infection in CHSE-214 fish cell line.

**Conclusions**: Despite not being infected, rainbow trout RBCs could respond to IPNV with increased expression of antiviral genes. Fish RBCs could be considered as mediators of the antiviral response and therefore targets of new strategies against fish viral infections. Further research is ongoing to completely understand the molecular mechanism that triggers this antiviral response in rainbow trout RBCs.

## Introduction

Fish viral infections cause significant losses in aquaculture. Infectious pancreatic necrosis (IPN) is a highly contagious viral disease with a high impact on salmonid aquaculture industry. Infectious pancreatic necrosis virus (IPNV) is the causative agent of IPN and was the first fish virus isolated in cell culture ^1^. IPNV outbreaks are usually related to high mortality rates in salmonid aquaculture, especially in young individuals ^2, 3^, highlighting the urgent necessity for the development of efficient strategies in vaccination. IPNV belongs to the *Aquabirnavirus* genus within the *Birnaviridae* family. Viruses of this family are non-enveloped particles with a double stranded RNA genome. This genome consists of two segments: the A segment contains the information to encode a protein that is post-translationally cleaved into VP2, VP3 and VP4 viral proteins; the B segment encodes the viral RNA polymerase VP1 ^4^. VP2 and VP3 are the major structural and immunogenic proteins, as they represent 64% of the total proteins of the virion ^5^.

In contrast to mammals, fish, reptiles and avian red blood cells (RBCs) are nucleated. Typically, the role associated with RBCs has been the transport of O _2_ to different tissues and gas exchange. However, a whole set of biological processes related to the immune response has been recently reported for nucleated RBCs from different species: recognition of pathogen associated molecular patterns ^6, 7^ through expression of pattern recognition receptors, such as toll-like receptors (TLRs) ^8^; production of cytokine-like factors ^7, 9– 11^; phagocytosis ^12^; and formation of complement immune complexes ^13^. Fish RBCs are known to be the target of some viruses, such as infectious salmon anemia virus (ISAV) ^11^ and piscine orthoreovirus (PRV) ^14, 15^. Furthermore, both viruses can induce immune responses in infected RBCs, characterized by the expression of genes related to IFN-1 (type I interferon) pathway. Besides, recently it has been shown that viral hemorrhagic septicemia virus (VHSV) halted replication in rainbow trout RBCs could induce cytokine production ^16^.

In view of the aforementioned evidence, this study was aimed to evaluate the immune response of rainbow trout RBCs against IPNV, one of the most ubiquitous viral fish pathogens. To achieve this objective, we first analyzed the infectivity of IPNV in rainbow trout RBCs. Then, RBCs immune response was evaluated after *ex vivo* exposure to IPNV, by means of antiviral gene and protein expression analysis. Finally, we evaluated the ability of RBCs to confer protection against IPNV in CHSE-214 cells, which are susceptible to IPNV infection. To summarize, here we report the regulation of the immune response of rainbow trout RBCs by IPNV, a non-infective virus in this cell type. This immune response was characterized by the expression of genes related to the IFN-1 pathway, Mx production and induction of an antiviral state to IPNV in CHSE-214 cells.

## Methods

### Animals

Rainbow trout ( *Oncorhynchus mykiss*) individuals of approximately 10 g were obtained from a commercial fish farm (PISZOLLA S.L., CIMBALLA FISH FARM, Zaragoza, Spain). Fish were maintained at the University Miguel Hernandez (UMH) facilities with a re-circulating dechlorinated-water system, at a stocking density of 1 fish/3L, at 14°C, and fed daily with a commercial diet (SKRETTING, Burgos, Spain). Fish were acclimatized to laboratory conditions over 2 weeks before experimentation. The number of fish used is indicated for each experiment/figure.

### RBCs purification

Rainbow trout were sacrificed by overexposure to tricaine methanesulfonate (Sigma-Aldrich, Madrid, Spain) at 0.2 g/L. Peripheral blood was sampled from the caudal vein using insulin syringes (NIPRO Bridgewater, NJ). Approximately 100 µL of blood was diluted in RPMI-1640 medium (Dutch modification) (Gibco, Thermo Fischer Scientific Inc., Carlsbad, CA) supplemented with 10% FBS (Cultek, Madrid, Spain), 1 mM pyruvate (Gibco), 2 mM L-glutamine (Gibco), 50 µg/mL gentamicin (Gibco), 2 µg/mL fungizone (Gibco) and 100 U/mL penicillin/streptomycin (Sigma-Aldrich). Then, RBCs were purified by two consecutive density gradient centrifugations with Histopaque 1077 (7206g, Ficoll 1.007; Sigma-Aldrich). Finally, RBCs were washed twice with RPMI 2% FBS. Purity of RBCs of 99.9% was estimated by optical microscopy evaluation. Then, purified RBCs were cultured in the above indicated medium at a density of 10 ^7^ cells/mL, in cell culture flasks, at 14°C, overnight.

### Viral infection assays

*Ex vivo* rainbow trout RBCs along with CHSE-214 cell line (Chinook Salmon Embryo, ATCC CRL-1681) were infected using IPNV Sp strain ^17^. IPNV was grown as previously described ^18^. *Ex vivo* RBCs exposure to IPNV was performed by incubating RBCs with diluted IPNV at the indicated MOI (multiplicity of infection) in RPMI 2% FBS. After three hours of incubation at 14°C, RBCs were centrifuged at 1600 rpm for 5 minutes and then washed with medium to completely eliminate the non-adsorbed excess of virus. Finally, RBCs were placed in 24 well plates (Corning Costar, Sigma-Aldrich, Madrid, Spain) with 500 µl of RPMI 2% FBS. The whole process was done at 14°C. Infection of the CHSE-214 cell line was done by incubating IPNV diluted in RPMI 2% FBS at the desired MOI for 1 hour at 14°C. After that, medium was removed and RPMI 2% FBS was added to each well. Infected CHSE-214 cells were maintained at 14°C ^18^.

In time course experiments, the initial supernatant with IPNV was not removed. When each of the time points was reached, RBCs were washed with cell culture medium and CHSE-214 cells with PBS supplemented with calcium.

### Viral titration assay

The virus titer in IPNV-exposed RBCs supernatants was quantified by TCID _50_ and by RT-qPCR. Briefly, different dilutions of the supernatants (from 10 ^-1^ to 10 ^-4^) were added to CHSE-214 cell monolayers, and incubated at 14°C for 90 minutes. Then, the virus was removed and infected CHSE-214 cell monolayers covered with a solution of RPMI 2% FBS. Cell plates were incubated at 14°C for 7 days. For RT-qPCR titration, 30 µL of IPNV with known titer (10 ^9^ TCID _50_/mL) and 30 µL of IPNV-exposed RBCs supernatants were used to extract RNA and synthetize cDNA, as explained hereafter. Ten-fold serial dilutions from 10 ^8^ to 10 ^2^ TCID _50_/mL were done to obtain IPNV cDNA and create a standard line.

### RNA isolation and DNAse treatment

The E.Z.N.A.® Total RNA Kit (Omega Bio-Tek Inc., Norcross, GA) was used for total RNA extraction, in accordance with manufacturer’s instructions. DNAse treatment was done in order to eliminate residual genomic DNA using TURBO™ DNase (Ambion, Thermo Fischer Scientific Inc.), following the manufacturer’s instructions. RNA was quantified with a NanoDrop® 377 Spectrophotometer (Nanodrop Technologies, Wilmington, DE).

### Gene expression by RT-qPCR

cDNA was synthesized from RNA using M-MLV reverse transcriptase (Invitrogen, Thermo Fischer Scientific Inc.), as previously described ^19^. Final concentration of cDNA was 6 ng/µL. RT-qPCR reactions were performed in a total volume of 20 μl using 12 ng of cDNA, 10 μl of TaqMan universal PCR master mix (Thermo Fischer Scientific), 900 nM final concentration of each primer (300 nM for IPNV segment A) and 300 nM of probe (150 nM for IPNV segment A). RT-qPCR was performed using the ABI PRISM 7300 System (Thermo Fischer Scientific). Cycling conditions were 50°C for 2 min and 95°C for 10 min, followed by 40 cycles at 95°C for 15 s and 60°C for 1 min.

Gene expression was analyzed by the 2 ^-ΔΔ^Ct method ^20^. The eukaryotic 18S rRNA gene (Cat#4310893E, Thermo Fischer Scientific) was used as endogenous control. Primers and probes are listed in Table 1.

**Table 1.** List of primers and probes.

Gene	Forward primer (5’ – 3‘)	Reverse primer (5’ – 3‘)	Probe (5’ – 3‘)	Reference or accession number
IPNV SA	TCTCCCGGGCAGTTCAAGT	CGGTTTCACGATGGGTTGTT	CCAGAACCAGGTGACGAGTATGAGGACTACAT	18
*tlr3*	ACTCGGTGGTGCTGGTCTTC	GAGGAGGCAATTTGGACGAA	CAAGTTGTCCCGCTGTCTGCTCCTG	NM_001124578.1
*irf7*	CCCAGGGTTCAGCTCCACTA	GGTCTGGCAACCCGTCAGT	TCGAGCCAAACACCAGCCCCT	AJ829673
*ifn1*	ACCAGATGGGAGGAGATATCACA	GTCCTCAAACTCAGCATCATCTATGT	AATGCCCCAGTCCTTTTCCCAAATC	AM489418.1
*mx1–3*	TGAAGCCCAGGATGAAATGG	TGGCAGGTCGATGAGTGTGA	ACCTCATCAGCCTAGAGATTGGCTCCCC	28
*pkr*	GACACCGCGTACCGATGTG	GGACGAACTGCTGCCTGAAT	CACCACCTCTGAGAGCGACACCACTTC	NM_001145891.1
*il8*	AGAGACACTGAGATCATTGCCAC	CCCTCTTCATTTGTTGTTGGC		29
*ifnγ*	CAAACTGAAAGTCCACTATAAGATCTCCA	TCCTGAATTTTCCCCTTGACATATTT		30
*tnfα*	AGCATGGAAGACCGTCAACGAT	ACCCTCTAAATGGATGGCTGCTT		31

### Antibodies

Several antibodies were used to stain cells for cytokines and to measure polypeptides in RBCs extracts by western blotting. They are briefly described below and their Research Resource Identifiers (RRIDs) given. For intracellular staining, mouse polyclonal antibodies against rainbow trout IL1β (RRID: AB_2716269) ^21, 22^, IL8 (RRID: AB_2716272) ^23^ and TNF-α (RRID: AB_2716270) ^24^ were produced at the laboratory of Dr. Luis Mercado. Rabbit polyclonal antibody against rainbow trout Mx3 (RRID: ABA_2716267) ^25, 26^ was produced at the laboratory of Dr. Amparo Estepa. Anti-IPNV-VP3 monoclonal antibody 2F12 (RRID: AB_2716296) was used for IPNV labelling ^27^. Anti-rabbit IgG (H+L) CF™ 488 antibody produced in goat and anti-mouse IgG (H+L) CF™ 488 antibody produced in goat were used as secondary antibodies for proteins and anti-mouse IgG (H+L) CF™ 647 produced in goat to detect 2F12 antibody.

For western blotting, rabbit polyclonal antibody against human eIF2α-P (Cat# E2152, RRID:AB_259283) and rabbit polyclonal antibody against human α-Actin (Cat#2066, RRID:AB_476693) were purchased from Sigma-Aldrich.

### Western blot

Control and IPNV-exposed RBCs pellets (≈10 ^7^ cells) were used for protein extraction. Cell pellets were washed 3 times with PBS and then resuspended in 30 µl of PBS with a cocktail of protease inhibitors (Sigma-Aldrich). Then, cells were frozen/thawed 3 times and lysed using an eppendorf micropistile (Eppendorf, Hamburg, Germany). Samples were loaded in Tris–Glycine sodium dodecyl sulfate 12% polyacrylamide gels under reducing conditions. Electrophoresis was performed at 200 V for 60 min. For blotting, the proteins in the gel were transferred for 80 min at 100 V in transfer buffer (2.5 mM Tris, 9 mM glycine, 20% methanol) to nitrocellulose membranes (BioRad, Madrid, Spain). Then, membranes were blocked with 8% dry milk and 1% Tween-20 in PBS and incubated with eIF2α-P or α-Actin antibodies, at the recommended dilutions in PBS containing 0.5% dry milk and 0.5% Tween-20 at 4°C and overnight. Incubation with secondary antibody GAR-Po (Sigma-Aldrich) was done in 0.5% milk 0.5% Tween-20 in PBS for 45 min. Membranes were washed 3 times with PBS containing 1% dry milk 0.5% Tween-20 for 15 min after every antibody incubation. Finally, the membrane was washed 3 times with PBS before analysis of the peroxidase activity. Peroxidase activity was detected using ECL chemiluminescence reagents (Amersham Biosciences, Buckinghamshire, UK) and revealed by exposure to X-ray. Protein bands from western blotting were analysed by densitometry using the *Scion Image 4.0.2* Software (RRID: SCR_008673) ( www.scionorg.com).

### Intracellular immunofluorescence stain and flow cytometry

RBCs were fixed with 4% paraformaldehyde (PFA; Sigma-Aldrich) and 0.08% glutaraldehyde (Sigma-Aldrich) diluted in RPMI medium for 20 minutes. Then, RBCs were incubated with permeabilization buffer containing 0.05% saponin (Sigma-Aldrich) in RPMI, for 15 minutes. Primary antibodies were used at 1/50 dilution for IL-1β, IL-8 and TNF-α, 1/300 for Mx and 1/500 for 2F12 in permeabilization buffer and incubated for 60 minutes at room temperature. Secondary antibodies were incubated for 30 minutes at 1/200 dilution. RBCs were washed with permeabilization buffer after antibody incubations. Finally, RBCs were kept in PFA 1% in PBS. For nuclear staining, RBCs were stained with 1 μg/mL of 4′-6-408 Diamidino-2-phenylindole (DAPI; Sigma-Aldrich) for 5 minutes. Flow cytometry (FC) analysis was done in a BD FACSCanto™ II (BD Biosciences) flow cytometer. Immunofluorescence (IF) images were performed in the INCell Analyzer 6000 Cell imaging system (GE Healthcare, Little Chalfont, UK).

### Antiviral activity of conditioned medium

Conditioned medium (CM) was obtained from control and IPNV-exposed RBCs at MOI 0.5, during 3 days. The CMs were clarified at 1600 rpm for 5 min. IPNV titer in the supernatants of IPNV-exposed RBCs resulted in 10 TCID _50_/mL or less, therefore viral presence in the supernatants was obviated. To test the antiviral activity of the CM, confluent CHSE-214 cells (7.8×10 ^4^ cells/well), seeded in 96 well plates, were pre-treated with 100 µL of each supernatant at the indicated dilutions for 24 hours. After that, CHSE-214 cells were infected, as described previously, with IPNV at MOI 0.05, for 24 hours. Finally, intracellular staining of IPNV foci was carried out.

### Intracellular staining of IPNV foci

CHSE-214 cells were fixed with PFA diluted at 4% in PBS followed by a second fixation with cold methanol. Each fixation step lasted 15 minutes. Cells were washed with PBS after each fixation step. Blocking buffer containing 5% goat serum (Sigma-Aldrich) and 0.3% Triton X-100 (Sigma-Aldrich) was added to each well with the cells for 1 hour. Then, anti-VP3 2F12 antibody was diluted 1/500 in antibody dilution buffer (1% BSA (Sigma-Aldrich), 0.3% Triton X-100) and was incubated for 1 hour. FITC-labelled goat anti-rabbit was used as secondary antibody at 1/300 dilution. Cells were washed three times after each antibody incubation with PBS. IF images were taken INCell Analyzer 6000 imaging system. *IN Cell Developer Toolbox 1.9.2* (RRID: SCR_015790; GE Healthcare, Little Chalfont, UK) was used to count number of IPNV foci (positive areas after image segmentation were selected when >21000 fluorescence units and >2500 µm ^2^ criteria was reached).

### MTT assays

Cell viability was tested using MTT (3-(4,5-dimethylthiazol-2-yl)-2,5-diphenyltetrazolium bromide) colorimetric assay ^32^. Briefly, 25 µl of MTT at a final concentration of 1.9 mg/mL were added to previously treated CHSE-214 cells monolayers, seeded in 96 well plates. Cells were incubated for 3 hours at 21°C with the reagent. Then, the medium was removed from the wells. Formazan crystals were dissolved in 100 µl of 100% DMSO, incubated for 30 minutes. Absorbance was read at 570 nm in the EON™ microplate spectrophotometer (Biotek, Winooski, VT). Percentage of viable cells was calculated as follows: absorbance treated cells/absorbance non-treated cells) x100.

### Software and statistics

All the figures and graphics show the mean and standard deviation of the data. P-values associated with each graphic are represented by the legends: *, p-value < 0.05; **, p-value < 0.01; ***, p-value < 0.001, ****, p-value < 0.0001. *Graphpad Prism 6* (RRID: SCR_002798, www.graphpad.com) (Graphpad Software Inc., San Diego, A) was used for preparing graphs and preforming statistical calculations. FC data were analyzed using *Flowing Software 2.5.1* (RRID: SCR_015781)( www.flowingsoftware.com) to obtain Mean Fluorescence Intensity (MFI) and Mean Relative Fluorescence Intensity (MRFI) (relative to control cells) values.

### Ethics approval

Methodology was carried out in accordance with the Spanish Royal Decree RD 53/2013 and EU Directive 2010/63/EU for animals used in research experimentation. All experimental protocols involving animal handling were also reviewed and approved by the Animal Welfare Body and the Research Ethics Committee at the Miguel Hernandez University (approval number 2014.205.E.OEP; 2016.221.E.OEP) and performed by qualified research personnel.

## Results

### IPNV did not infect rainbow trout RBCs

**Figure 1.**
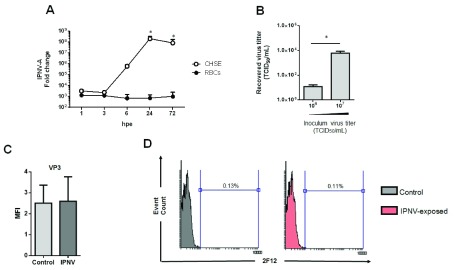
Infectivity of IPNV in RBCs. ( **A**) Time-course experiment of the expression of IPNV segment A (IPNV-A) in RBCs (●) (n = 6) and CHSE-214 cells (○) (n = 2) at MOI 0.5. Data is represented as mean±SD. Kruskal-Wallis Test with Dunn´s Multiple Comparison post-hoc test was performed among all time-points post-exposure in comparison with control time point (0 hpi) (*, p-value < 0.05). ( **B**) Recovered virus titer in supernatants from IPNV-exposed RBCs with an inoculum titer of 10 ^6^ (MOI 0.5) and 10 ^7^ (MOI 5) TCID _50_/mL obtained after 72 hpe (n = 5). Data is represented as mean±SD. Mann-Whitney test was performed among both conditions (*, p-value < 0.05). ( **C**) MFI (mean fluorescence intensity) of viral protein VP3 in control and IPNV-exposed RBCs at MOI 0.5 and 3 dpe (n = 6) Mann-Whitney test was performed among both conditions. ( **D**) Representative flow cytometry histograms of IPNV VP3 protein detection in control and IPNV-exposed RBCs at MOI 0.5 and 3 dpi.

To evaluate the infectivity of IPNV in rainbow trout RBCs, RBCs were exposed to IPNV at MOI 0.5 and the viral RNA was evaluated by RT-qPCR in the cell pellet at different times post-exposure. IPNV infectivity was also evaluated in parallel in the CHSE-214 cell line, used as a positive control of infection. IPNV segment A (IPNV-A) RNA levels inside RBCs and CHSE-214 cell line were similar at 1 and 3 hours post-exposure (hpe) ( Figure 1A). After 6 hpe, IPNV-A RNA level was 3 logarithms lower in RBCs in comparison with CHSE-214 cells. On the other hand, the titer of IPNV in the supernatants from IPNV-exposed RBCs at a MOI of 0.5 and 5, was evaluated by TCID _50_, at 3 days post-exposure (dpe), and showed a recovered titer of 5 and 4 logarithms lower, respectively ( Figure 1B). Furthermore, the supernatants titrated by RT-qPCR, were below the lowest limit of detection 10 ^2^ TCID _50_ ( Table 2). Moreover, FC analysis of control and IPNV-exposed RBCs for IPNV VP3 protein did not show significant differences ( Figure 1C and D). Therefore, IPNV did not infect rainbow trout RBCs.

**Table 2.** Rt-qPCR virus titration. Ct value ± standard deviation from standard line points (10 ^8^ to 10 ^2^ dilutions) and supernatants from IPNV-exposed RBCs at MOI 0.5, at 3 and 6 dpe. (n=7 individuals).

Sample	Ct value ± SD
10 ^8^ TCID _50_	25,885 ± 0,052
10 ^7^ TCID _50_	29,856 ± 0,117
10 ^6^ TCID _50_	33,165 ± 0,168
10 ^5^ TCID _50_	36,057 ± 0,11
10 ^4^ TCID _50_	39,126 ± 0.873
10 ^3^ TCID _50_	Undetected
10 ^2^ TCID _50_	Undetected
RBCs #1 3 dpe	Undetected
RBCs #1 6 dpe	Undetected
RBCs #2 3 dpe	Undetected
RBCs #2 6 dpe	Undetected
RBCs #3 3 dpe	Undetected
RBCs #3 6 dpe	Undetected
RBCs #4 3 dpe	Undetected
RBCs #4 6 dpe	Undetected
RBCs #5 3 dpe	Undetected
RBCs #5 6 dpe	Undetected
RBCs #6 3 dpe	Undetected
RBCs #6 6 dpe	Undetected
RBCs #7 3 dpe	Undetected
RBCs #7 6 dpe	Undetected
NTC	Undetected

### IPNV exposure increased the expression of interferon-related antiviral genes and proteins in rainbow trout RBCs

**Figure 2.**
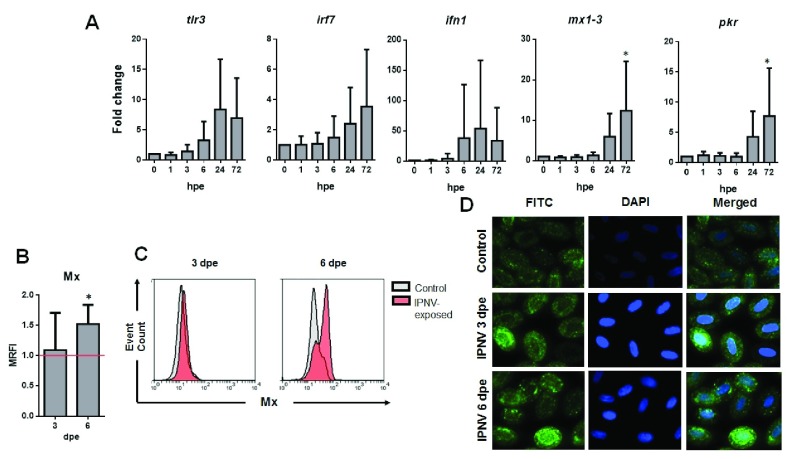
RBCs IFN-related antiviral response against IPNV. ( **A**) Gene expression of *tlr3*, *irf7*, *inf1*, *mx1–3* and *pkr* in IPNV-exposed RBCs at the indicated times post-infection and MOI 0.5, measured by RT-qPCR. Data represent mean±SD (n = 6). Kruskal-Wallis Test with Dunn´s Multiple Comparison post-hoc test was performed among all time-points post-exposure in comparison with control time point (0 hpi) (*, p-value < 0.05). ( **B**) Mx protein MRFI (mean relative fluorescent intensity, relative to control cells) in IPNV-exposed RBCs at MOI 0.5 (n = 5). ( **C**) Flow cytometry histograms of Mx protein expression from control (grey) and IPNV-exposed (red) RBCs at MOI 0.5 and the indicated days post-exposure (dpe). ( **D**) Representative immunofluorescence images of Mx protein expression in control and IPNV-exposed RBCs at MOI 0.5 (FITC – Mx protein expression, DAPI - Nuclei) (IF representative of 40 images).

To determine if IPNV would induce an antiviral response in RBCs, RT-qPCR analysis of IFN-related antiviral genes was performed for IPNV-exposed RBCs. The results showed that *mx1–3* and *pkr* genes were significantly expressed at 72 hpe. On the other hand, *ifn1* gene presented a tendency to increase its expression after 6 hpe, having a peak at 24 hpe. Also, *tlr3* gene expression tended to be upregulated at 24 hpe, whereas *irf7* expression was upregulated at 72 hpe ( Figure 2A). Three and six dpe with IPNV, RBCs were stained intracellularly with an anti-Mx antibody and analyzed by FC and immunofluorescence imaging (IF). The results showed a significant increment in the expression of Mx protein at 6 dpe by both FC an IF ( Figure 2B and D). FC histograms showed, at 6 dpe, that RBCs depicted distinct peaks of Mx expression, showing that the expression of Mx in RBCs was heterogeneous in the total RBCs population ( Figure 2C).

### Conditioned medium from IPNV-exposed RBCs protected CHSE-214 cells against IPNV infection

**Figure 3.**
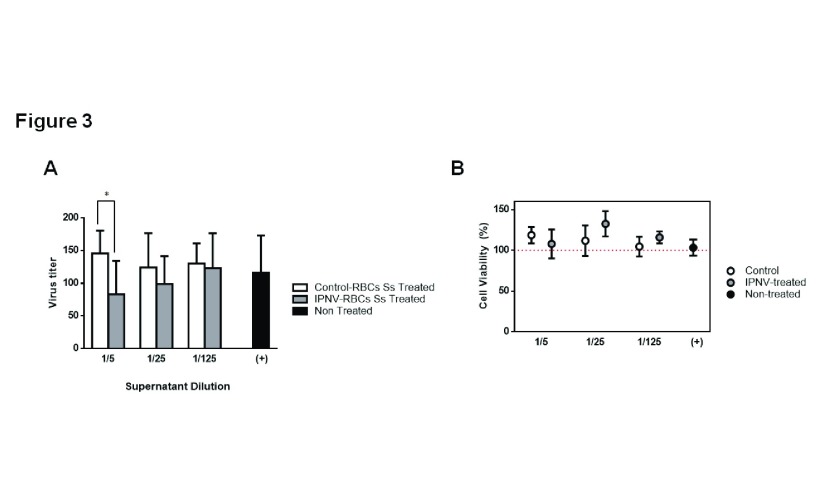
Antiviral activity of the conditioned media from IPNV-exposed RBCs. ( **A**) Viral titers (FFU/mL) in CHSE-214 cells infected with IPNV at MOI 0.05 previously non-treated (black) or treated with either supernatants from control RBCs (white) or IPNV-exposed RBCs (grey), during 24 hours, at the indicated dilutions (n = 4, performing triplicates from each individual). Two-way ANOVA, with Sidak´s multiple comparison test, was performed among the different dilutions and conditions to test statistical differences. ( **B**) Percentage of viable CHSE-214 cells pre-treated with conditioned medium from control and IPNV-exposed RBCs, during 24 hours, and relative to non-treated CHSE-214 cells. Percentage of viable cells was calculated as follows: absorbance treated cells/absorbance non-treated cells) x100.

To analyze if IPNV-exposed RBCs could secrete factors that were capable to protect other fish cells against IPNV infection, conditioned medium (CM) from control and IPNV-exposed RBCs (with IPNV titer <10 TCID _50_/mL) were added to CHSE-214 cells prior to infection. Figure 3A shows a significant decrease in the number of IPNV infective focus forming units (FFU/mL) when pre-treating with 1/5 diluted CM from IPNV-exposed RBCs. CHSE-214 cells viability, by means of an MTT colorimetric assay, was not affected by the exposure to CM ( Figure 3B).

### IPNV exposure decreased the expression of cytokines in rainbow trout RBCs

**Figure 4.**
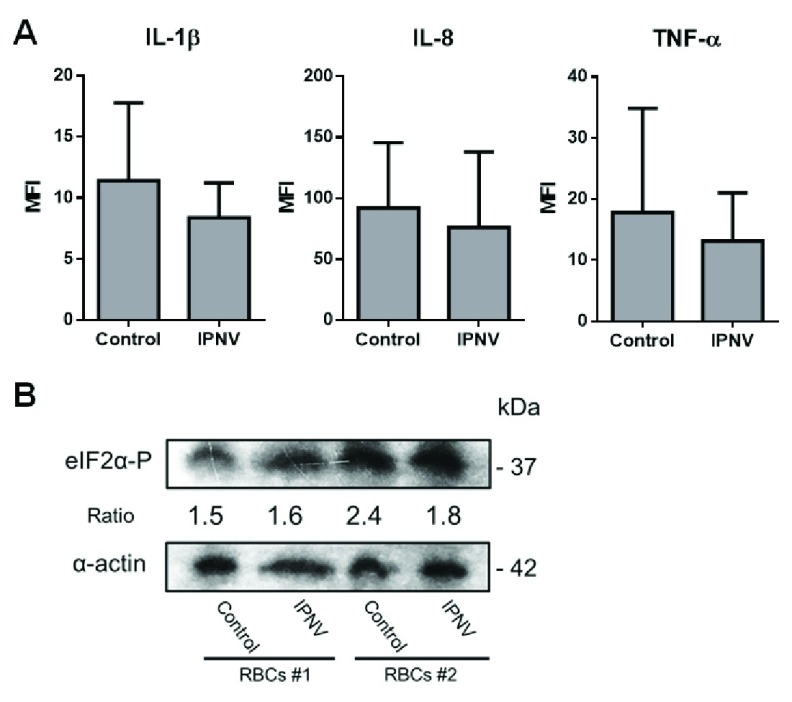
IPNV-exposure decreased cytokine levels in rainbow trout RBCs. ( **A**) Intracellular MFI (mean fluorescent intensity) values of IL-1β, IL-8 and TNFα from control and IPNV-exposed RBCs at MOI 0.5 and 3 dpe measured by FC (flow cytometry)(n = 6). Mann-Whitney test was performed among both conditions. ( **B**) Phosphorylation of translation initiation factor eIF2α in IPNV-exposed RBCs. Representative western blot of eIF2α-P in control and IPNV-exposed RBCs from two individuals at MOI 0.5, 3 dpe. Densitometry ratios were done relativizing to α-actin. Mann-Whitney test was performed among both conditions.

To evaluate whether *ex vivo* rainbow trout RBCs could produce cytokines in response to IPNV exposure, RBCs were exposed to IPNV and IL-1 β, IL-8 and TNF-α protein levels were evaluated by means of FC and IF in control and IPNV-exposed cell cultures. The results showed a decrease in the protein expression of IL-1β, IL-8 and TNFα in IPNV-exposed RBCs ( Figure 4A).

### IPNV exposure did not induce phosphorylation of the α-subunit of the eukaryotic translational initiation factor 2 (eIF2α) in rainbow trout RBCs

The phosphorylation of the translation initiation factor eIF2α is a key mechanism of global inhibition of translational initiation ^33^ and it has been described to happen after IPNV infection in the permissive cell line CHSE-214 cells ^34^. In this sense, since IPNV-exposed RBCs depicted a small downregulation of the evaluated cytokines protein levels, we further investigated whether IPNV exposure could reduce protein translation in RBCs by triggering the phosphorylation of eIF2α. However, the results revealed no changes in the phosphorylation of eIF2α ( Figure 4B).

Excel file containing qPCR data Each sheet contains the raw Ct values for the indicated figure numbers, organized by samples (rows) and genes (columns). Click here for additional data file. Copyright: © 2017 Nombela I et al. 2017 Data associated with the article are available under the terms of the Creative Commons Zero "No rights reserved" data waiver (CC0 1.0 Public domain dedication).

Excel file containing the virus titration data Contains the virus titer (TCID _50_/mL) results of the indicated figure number. Click here for additional data file. Copyright: © 2017 Nombela I et al. 2017 Data associated with the article are available under the terms of the Creative Commons Zero "No rights reserved" data waiver (CC0 1.0 Public domain dedication).

Flow cytometry data Each folder contains the Flow Cytometry Standard (.fcs) format files. Source data files are organized by figure number, and then by sample number, condition and antibody. Click here for additional data file. Copyright: © 2017 Nombela I et al. 2017 Data associated with the article are available under the terms of the Creative Commons Zero "No rights reserved" data waiver (CC0 1.0 Public domain dedication).

Excel file containing the Focus Forming Units (FFU) counting for Figure 3A Click here for additional data file. Copyright: © 2017 Nombela I et al. 2017 Data associated with the article are available under the terms of the Creative Commons Zero "No rights reserved" data waiver (CC0 1.0 Public domain dedication).

Excel file containing MTT absorbance raw data Click here for additional data file. Copyright: © 2017 Nombela I et al. 2017 Data associated with the article are available under the terms of the Creative Commons Zero "No rights reserved" data waiver (CC0 1.0 Public domain dedication).

Excel file containing the densitometry raw data of eIF2α-P and α-Actin western blots Related uncropped blots are included. Click here for additional data file. Copyright: © 2017 Nombela I et al. 2017 Data associated with the article are available under the terms of the Creative Commons Zero "No rights reserved" data waiver (CC0 1.0 Public domain dedication).

## Discussion

Previously, we have demonstrated that rainbow trout RBCs can respond to VHSV, a ssRNA virus not targeting RBCs, halting its replication, downregulating type I interferon-related genes, showing global protein downregulation in the cell and phosphorylation of the translation initiation factor eIF2α ^16^.

It is known that IPNV primarily targets pancreatic and liver cells ^35^. It has been also reported that IPNV was detectable in kidney hematopoietic tissue, corpuscles of Stannius, in Islets of Langherhans, in the lamina propria of the pyloric caeca, the gill arch connective tissue, the ventricle of the heart and dermis of the skin ^35^. Our results showed that IPNV did not replicate in RBCs, although small amounts of IPNV were persistently found inside RBCs after 3 dpe (≈ 10 ^3^ TCID _50_/mL). Similarly, IPNV has been shown to enter mammalian cells, without significant levels of replication, being this entry suggested to be receptor mediated ^36^. From our results, the persistence of IPNV in RBCs after 72 hpe could point out the entry of the virus inside RBCs. However, we could not further verify the presence of the IPNV inside RBCs ( Figure 1).

Nevertheless, despite the lack of replication of IPNV in RBCs, IPNV could induce an antiviral gene expression mediated by the IFN pathway, as it has been observed in RBCs productive infections with ISAV ^11^ and PRV ^14^. As shown by our results, *ifn1* and IFN-1 related genes ( *irf7*, *pkr* and *mx*) expression levels were increased time-dependently in response to IPNV-exposure. High inter-individual variability was observed, similarly to that found in the RBCs from salmons challenged with PRV ^37^. In addition, although we could not verify the entry and uncoating of IPNV inside RBCs, we could observe an increment in the expression of the *tlr3* gene in parallel to the expression of the other IFN-related genes in IPNV-exposed RBCs. This could indicate the activation of the TLR3/IFN pathway by the presence of intracellular viral dsRNA.

IFN-1 leads to the expression of interferon stimulated genes (ISGs) ^38^. Among ISGs, the antiviral protein Mx has a well characterized antiviral role. Confirming those expectations, our results showed the significant upregulation of the Mx protein 6 dpe, after having a peak of its gene expression at 3 dpe. Previously, a positive correlation between the expression of Mx protein and the inhibition of IPNV in CHSE-214 cells has been established ^39^. Therefore, Mx protein production in IPNV-exposed RBCs could be involved in the low IPNV titers observed. The high basal levels of Mx protein detected inside RBCs ( Figure 2D), much elevated than those for CHSE-214 cells ( Figure S1), could be implicated in the early disappearance of IPNV inside RBCs. A similar hypothesis has been made in the abortive infection of VHSV in the RTS-11 cell line ^40^ and in rainbow trout RBCs ^16^, where upregulation or high constitutive expression of *mx* genes was speculated to be related to the inhibition of the virus.

Moreover, our results showed that CM from RBCs exposed to IPNV could partially protect CHSE-214 cells from IPNV infection. Similar to other cell types, this antiviral activity has been also observed in CM of RTS11 and RTG-2 cells exposed to Poly (I:C) (polyinosinic:polycytidylic acid) and/or infected with chum salmon reovirus ^41^. The fact that RBCs can secrete factors that confer protection against IPNV infection in other cell lines could indicate that RBCs, despite not being permissive to IPNV infection, may exhibit an antiviral response. Besides, we evaluated the production of cytokines in IPNV-exposed RBCs. Previously, the expression of IL-1β in salmon gill and head kidney tissues ^42^, IL-8 in rainbow trout head kidney tissue ^43 ^ and TNFα in zebrafish embryonic cells ^44^ have been implicated in the immune response against IPNV; therefore, we chose these cytokines to evaluate the immune response of rainbow trout RBCs to IPNV exposure. However, our results showed a reduction trend of these proteins in IPNV-exposed RBCs.

A shutdown in protein synthesis by phosphorylation of eIF2α has been reported in CHSE-214 cells infected with IPNV ^34^. So far, in rainbow trout RBCs exposed to IPNV, although a trend to cytokine protein reduction was observed, no phosphorylation of eIF2α was detected and Mx protein expression was increased. IFN-1 has been reported to inhibit the production of IL-1β ^45^, therefore, the cytokine reduction trend observed could have been a result of the related IFN-1 pathway upregulation. In contrast, in rainbow trout RBCs, VHSV rhabdovirus induced phosphorylation of eIF2α and a cell shut-off characterized by the downregulation of the proteome ^16^.

Further studies are needed to completely understand the molecular mechanism through which IPNV triggers this immune response in rainbow trout RBCs. However, the lack of commercial antibodies against fish proteins involved in cell signaling networks limits the study of this area. The implication of RBCs during *in vivo* IPNV infection and the response against different strains of IPNV remains to be evaluated.

Finally, one of the potential applications of these results is that fish RBCs could be considered mediators of the antiviral response and therefore targets of novel DNA vaccines and of new strategies against fish viral infections.

## Data availability

The data referenced by this article are under copyright with the following copyright statement: Copyright: © 2017 Nombela I et al.

Data associated with the article are available under the terms of the Creative Commons Zero "No rights reserved" data waiver (CC0 1.0 Public domain dedication). 

**Dataset 1. Excel file containing qPCR data.** Each sheet contains the raw Ct values for the indicated figure numbers, organized by samples (rows) and genes (columns). doi, 10.5256/f1000research.12994.d182842 ^46^

**Dataset 2. Excel file containing the virus titration data.** Contains the virus titer (TCID _50_/mL) results of the indicated figure number. doi, 10.5256/f1000research.12994.d182843 ^47^

**Dataset 3. Flow cytometry data.** Each folder contains the Flow Cytometry Standard (.fcs) format files. Source data files are organized by figure number, and then by sample number, condition and antibody. doi, 10.5256/f1000research.12994.d182844 ^48^

**Dataset 4. Excel file containing the Focus Forming Units (FFU) counting for Figure 3A.** doi, 10.5256/f1000research.12994.d182845 ^49^

**Dataset 5. Excel file containing MTT absorbance raw data.** doi, 10.5256/f1000research.12994.d182846 ^50^

**Dataset 6. Excel file containing the densitometry raw data of eIF2α-P and α-Actin western blots.** Related uncropped blots are included. doi, 10.5256/f1000research.12994.d182847 ^51^

## Supplementary material

**Figure S1. qPCR Amplification plot of mx1-3 gene expression in RBCs and CHSE-214 cells.**
