# Supplementary figures and images for: Infectious pancreatic necrosis virus triggers antiviral immune response in rainbow trout red blood cells, despite not being infective
Source: F1000Res. 2017 Dec 13;6:1968. Originally published 2017 Nov 7. [Version 2] doi: 10.12688/f1000research.12994.2 (PMC5747336; doi:10.12688/f1000research.12994.2)

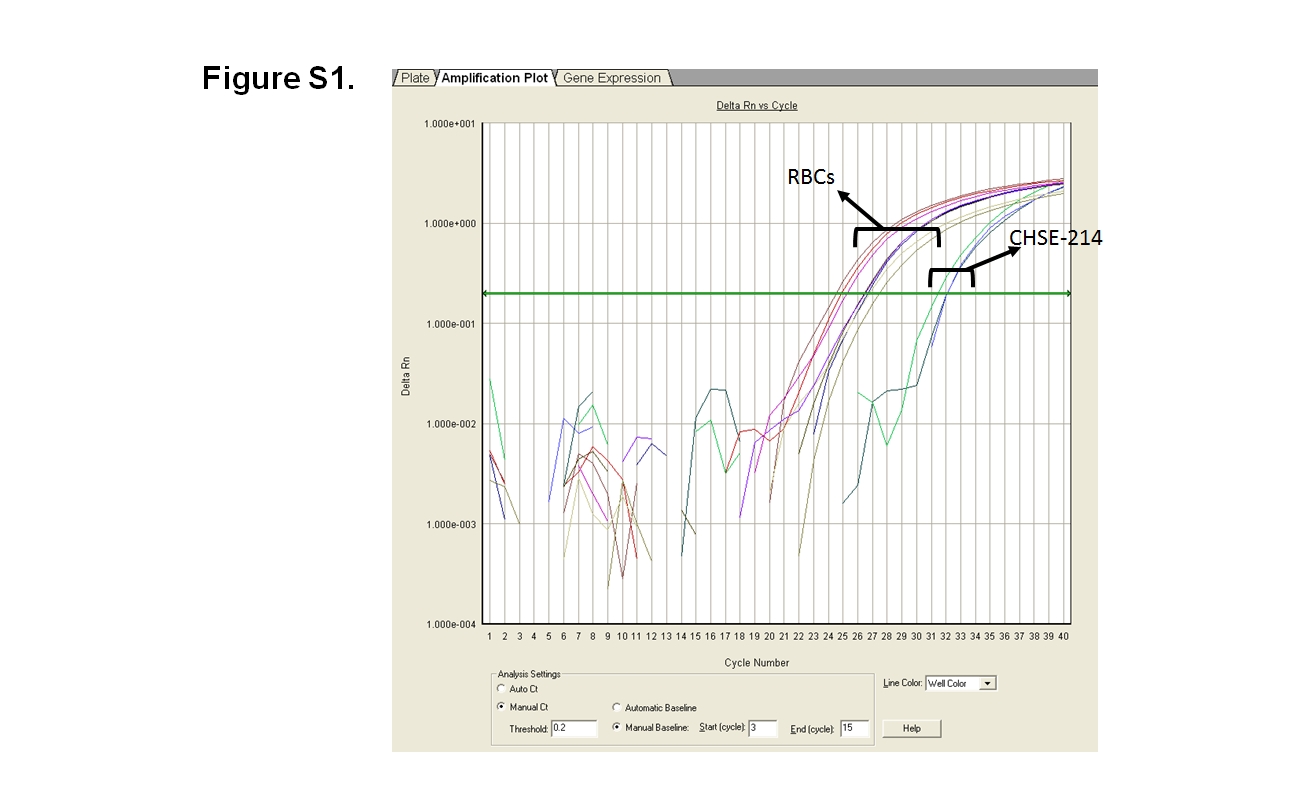

Supplement: Supplementary file 7 [file f1000research-6-14494-s0006.tgz › d942d7a8-8ba7-4aa7-a545-272ea1a85fc3.jpg]
