# Supplementary material for: Infectious pancreatic necrosis virus triggers antiviral immune response in rainbow trout red blood cells, despite not being infective
Source: F1000Res. 2017 Dec 13;6:1968. Originally published 2017 Nov 7. [Version 2] doi: 10.12688/f1000research.12994.2 (PMC5747336; doi:10.12688/f1000research.12994.2)
Supplement: Peer review history

# Peer review: Referee response for version 2

**DOI:** 10.5256/f1000research.14494.r29012

**Published:** 2017-12-21

**Authors:** Espen Rimstad, Maria Dahle

**Affiliations:** 1 Department of Food Safety and Infection Biology, Norwegian University of Life Sciences, Oslo, Norway; 2 Norwegian Veterinary Institute, Oslo, Norway

The authors have responded well to each individual point raised. We would recommend that fish populations used in experimental infections including immunological studies are routinely screened for pathogens before entering experimental infections, and not just assessed by measuring mortality. The lack of mortality may not warrant freedom of infection. The presence of low virulent variants of virus may be missed, but they could still have impact on parameters used for measuring immune responses.

We have read this submission. We believe that we have an appropriate level of expertise to confirm that it is of an acceptable scientific standard.

# Peer review: Referee response for version 1

**DOI:** 10.5256/f1000research.14090.r27697

**Published:** 2017-11-23

**Authors:** Niels C. Bols

**Affiliations:** 1 Department of Biology, University of Waterloo, Waterloo, ON, Canada

The manuscript by Maria de Mar Ortega-Villaizan Romo provides evidence that trout red blood cells can express antiviral mechanisms.  The discovery is very intriguing, as red blood cells appear to have addition functions beyond carry gases, and this might open up new ways to enhance fish health.  Therefore, I recommend indexing. As documented below, I do have many minor suggestions for improving the clarity and aesthetics of the manuscript.

**ABSTRACT**

Background

 Remove “highly”

 I suggest “However, the roles of RBCs in birnavirus….”

Methods:

 Remove “induced”

Results:

 Remove “-exposed RBCs”

Conclusions:  I suggest recasting a few sentences as shown below.

 “Despite not being infected, trout RBCs respond to IPNV with increased expression of antiviral genes.”

 In the last sentence, I suggest “that triggers this antiviral response in trout RBCs”

**INTRODUCTION**

*1 ^st^ paragraph  *2 ^nd^ line remove “Among all of the viral diseases,”

 It sounds awkward and the point being made is made again in the 4 ^th^ paragraph.

*2 ^nd^ paragraph*

 Remove “This implies that nucleated RBCs can support viral replication.”  Over 40 years ago, enucleated cells were shown to support replication of some viruses ^1^. Therefore, having a nucleus in RBCs might have nothing to do with their capacity to support the replication of some viruses.  For example, human RBCs might also have roles in viral infections.  Therefore, the first 2 sentences of this paragraph could be eliminated and the next sentence, after removing “for example”, would become the topic sentence.

*3 ^rd^ paragraph*

 Remove the sentence that begins “Recently, it has been shown that viral hemorrhagic septicemia virus (VHSV) can induce ….”

 I don’t see how it fits the subject of the paragraph or point out in the sentence that RBCs as well as stromal cells were studied.

*4 ^th^ paragraph*

 recast the last 2 sentences

 “However, we report that even though not infecting RBCs, IPNV induces in them the expression of antiviral genes in the INF-1 pathway.”  I am not sure that I would emphasize so strongly “protection against IPNV infection in CHSE-214 cells”.  This appears to be based on the 1/5 bar in Figure 3A.  I would like to have seen if protection also arose from 1 to 3 supernatant dilution or 1 to 10 supernatant dilution.  If either one or both of these dilutions did protect, it would strongly indicate that protection can be repeatability demonstrated.

**METHODS**

*Animals*

 Space between 1 and fish.

 Space after Spain)

*RBC purification*

 Was no anticoagulant used in the collection of the blood?  Please clarify.  Would thrombocytes be present among the RBCs?

 In the 3 ^rd^ last sentence I wonder if something should follow the 2%.  RPMI with 2 % FBS? This also comes in the next section.  Maybe define it here as RPMI with 2 % FBS (RPMI/FBS) and use in RPMI/FBS in subsequent section.

*Viral infection assays*

 No need to repeat *Oncorhynchus mykiss* here as the genus species name are mentioned earlier.

 In the 2 ^nd^ last sentence, I believe that the RPMI was “removed” rather than “retired”.

 Were RBC infected at 14 °C like CHSE-214 were?

*Viral titration assay*

 In the first sentence, “supernatants were quantified by TCID50 and by RT-qPCR.”

*RNA isolation and DNAse treatment*

 no suggestions

*Gene expression by RT-qPCR*

 “synthesized”

*Antibodies*

 I suggest the following topic sentence for this section.

 “Several antibodies were used to stain cells for cytokines and to measure polypeptides in RBC extracts by western blotting and they are briefly described below and their Research Resource Identifiers (RRIDs) given.”

*Western blot*

 no suggestions

*Intracellular immunofluorescence staining and flow cytometry * 

 no suggestions

*Antiviral activity of conditioned medium*

 Would not the conditioned medium (CM) have virus that had not bound to RBC and thus not pelleted at 1600 rpm and so was in the CM?  Please briefly discuss.

 Is ‘staining’ rather than “stain” meant in the following sentence and the next heading?

 “Finally, intracellular stain of IPNV foci was carried out.”

*Intracellular stain of IPNV foci*

 Add to the end of the 3 ^rd^ sentence “for 1 hour with the cells”.

*MTT assays*

 At the end of this section, a statement of how the absorbance readings are converted to percent viability should be given.  I know that it is mentioned in the legend for Figure 3.

*Software and statistics*

 I suggest the following modification to the 2 ^nd^ sentence.

 “was used for preparing graphs and preforming statistical calculations”.

*Ethics approval*

 no suggestions

**RESULTS** 

*IPNV did not infect trout RBCs*

 I suggest the following recasting of the last sentence and addition of a new last sentence.

 “Moreover, flow cytometry analysis of control and IPNV-exposed RBCs for IPNV VP3 protein did not show significant differences (Figure 1C and D).  Therefore, IPNV did not infect trout RBCs.”

*IPNV exposure increased the expression of interferon-related antiviral immune genes and proteins in RBCs*

 The first sentence should be shortened as follows

 “to determine if IPNV would induce an antiviral response in RBCs.”

 Are there antiviral genes that are not immune genes?

 In other words, can the description be simplified to just ‘antivrial genes”?

**DISCUSSION**

*2 ^nd^ paragraph*

 The last two sentences could refer to the data of the ms for support.

*4 ^th^ paragraph*

 I wonder if the authors could briefly discuss the apparent constitutive expression of Mx in RBCs as seen in Panel D of Figure 2.  Also could Mx be released from RBCs?  In cattle Mx1 is found in exosomes (see Racicot K et al., 2012, Am J Reprod Immunol) and exosomes can be released by RBCs (see Danesh A et al., 2014, Blood).

**FIGURES AND LEGENDS**

 Figure 1

 In panel A write out hpe on the X axis.

 Figure 2

 Is the staining in the control of interest or just background?  I mentioned this earlier under the Discussion.  Could any of the cells in panel D be thrombocytes?

 Figure 3

 Could FFU be written out here in the legend so figure stands alone?  The legend mentions doing a two-way ANOVA but no mention of a post test is given, although the 1/5 supernatant dilution is identified with an asterisk?

 Figure 4  Title legend has a spelling mistake and is a bit misleading because panel B is not covered by the current title.  I suggest the following title.

 “Reduction in RBC cytokine levels by IPNV and a possible mechanism”

 The legend should give more information so the figure can better stand by itself.

 Write out MFI (Mean Fluorescence Intensity) and FC (Flow Cytometry).

 At two places in the legend, mention is made of the Mann-Whitney test but the outcome is not clearly stated.

I have read this submission. I believe that I have an appropriate level of expertise to confirm that it is of an acceptable scientific standard.

## References

[1] FollettEAPringleCRWunnerWHSkehelJJ: Virus replication in enucleate cells: vesicular stomatitis virus and influenza virus. *J Virol*.1974;13(2) :394-9 435929910.1128/jvi.13.2.394-399.1974PMC355309

# Response

**Published:** 2017-12-01

**Authors:** Maria del Mar Ortega-Villaizan Romo

**Affiliations:** Universidad Miguel Hernandez, Spain

Dear Dr. Niels Bols,

Thank you very much for your positive review and suggestions for the manuscript. We have included your corrections and suggestions in the new version of the manuscript.

**ABSTRACT**

Background

Remove “highly”

Author´s response: It has been corrected.

I suggest “However, the roles of RBCs in birnavirus….”

Author´s response: It has been corrected.

Methods:

Remove “induced”

Author´s response: It has been corrected.

Results:

Remove “-exposed RBCs”

Author´s response: It has been corrected.

Conclusions:  I suggest recasting a few sentences as shown below.

“Despite not being infected, trout RBCs respond to IPNV with increased expression of antiviral genes.”

In the last sentence, I suggest “that triggers this antiviral response in trout RBCs”

Author´s response: It has been corrected.

**INTRODUCTION**

*1* *^st^* *paragraph  *2 ^nd^ line remove “Among all of the viral diseases,”

It sounds awkward and the point being made is made again in the 4 ^th^ paragraph.

Author´s response: It has been corrected.

*2* *^nd^* *paragraph*

Remove “This implies that nucleated RBCs can support viral replication.”  Over 40 years ago, enucleated cells were shown to support replication of some viruses ^1^. Therefore, having a nucleus in RBCs might have nothing to do with their capacity to support the replication of some viruses.  For example, human RBCs might also have roles in viral infections.  Therefore, the first 2 sentences of this paragraph could be eliminated and the next sentence, after removing “for example”, would become the topic sentence.

Author´s response: We have removed these sentences.

*3* *^rd^* *paragraph*

Remove the sentence that begins “Recently, it has been shown that viral hemorrhagic septicemia virus (VHSV) can induce ….”

I don’t see how it fits the subject of the paragraph or point out in the sentence that RBCs as well as stromal cells were studied.

Author´s response: We have changed this sentence to: “Besides, recently it has been shown that viral hemorrhagic septicemia virus (VHSV) halted replication in rainbow trout RBCs could induce cytokine production” as another reference of immune response by RBCs against virus.

*4* *^th^* *paragraph*

recast the last 2 sentences

“However, we report that even though not infecting RBCs, IPNV induces in them the expression of antiviral genes in the INF-1 pathway.”  I am not sure that I would emphasize so strongly “protection against IPNV infection in CHSE-214 cells”.  This appears to be based on the 1/5 bar in Figure 3A.  I would like to have seen if protection also arose from 1 to 3 supernatant dilution or 1 to 10 supernatant dilution.  If either one or both of these dilutions did protect, it would strongly indicate that protection can be repeatability demonstrated.

Author´s response: The protection conferred by the CM from IPNV-exposed RBCs diminished as the dilution increased from 1/5 to 1/25 and 1/125. However, we have not tried 1/3 or 1/10 dilutions. As Dr. Bols pointed out, it would have been interesting to check with lower dilution ranges. In relation to repeatability, this assay was done using the supernatants from 4 ex vivo infections, in triplicate each of them. Therefore, the repeatability could have been demonstrated by the assay itself?

We have rewritten the sentence to do not emphasize so strongly “protection” as follows: “the induction of an antiviral state to IPNV in CHSE-214 cells”.

**METHODS**

*Animals*

Space between 1 and fish.

Space after Spain)

Author´s response: It has been corrected.

*RBC purification*

Was no anticoagulant used in the collection of the blood?  Please clarify.  Would thrombocytes be present among the RBCs?

Author´s response: We did not use any anti-coagulant. The blood was immediately diluted in RPMI 10%FBS and slightly resuspended. In this way, no coagulation occurs. On the other hand, thrombocytes were eliminated with the double Ficoll purification.

In the 3 ^rd^ last sentence I wonder if something should follow the 2%.  RPMI with 2 % FBS? This also comes in the next section.  Maybe define it here as RPMI with 2 % FBS (RPMI/FBS) and use in RPMI/FBS in subsequent section.

Author´s response: It has been corrected.

*Viral infection assays*

No need to repeat *Oncorhynchus mykiss* here as the genus species name are mentioned earlier.

In the 2 ^nd^ last sentence, I believe that the RPMI was “removed” rather than “retired”.

Were RBC infected at 14 °C like CHSE-214 were?

Author´s response: It has been corrected and 14 °C added to RBCs infection protocol.

*Viral titration assay*

In the first sentence, “supernatants were quantified by TCID50 and by RT-qPCR.”

Author´s response: It has been corrected.

*RNA isolation and DNAse treatment*

no suggestions

*Gene expression by RT-qPCR*

“synthesized”

Author´s response: It has been corrected.

*Antibodies*

I suggest the following topic sentence for this section.

“Several antibodies were used to stain cells for cytokines and to measure polypeptides in RBC extracts by western blotting and they are briefly described below and their Research Resource Identifiers (RRIDs) given.”

Author´s response: We have included this sentence.

*Western blot*

no suggestions

*Intracellular immunofluorescence staining and flow cytometry * 

no suggestions

*Antiviral activity of conditioned medium*

Would not the conditioned medium (CM) have virus that had not bound to RBC and thus not pelleted at 1600 rpm and so was in the CM?  Please briefly discuss.

Author´s response: The supernatants of RBCs exposed to IPNV were titrated and resulted in approximately 10 TCID50/mL or less. We have added the following sentence to clarify this item: “IPNV titer in the supernatants of IPNV-exposed RBCs resulted in 10 TCID50/mL or less, therefore viral presence in the supernatants was obviated”.

Is ‘staining’ rather than “stain” meant in the following sentence and the next heading?

“Finally, intracellular stain of IPNV foci was carried out.”

Author´s response: It has been corrected.

*Intracellular stain of IPNV foci*

Add to the end of the 3 ^rd^ sentence “for 1 hour with the cells”.

Author´s response: It has been corrected.

*MTT assays*

At the end of this section, a statement of how the absorbance readings are converted to percent viability should be given.  I know that it is mentioned in the legend for Figure 3.

Author´s response: The formula has been added.

*Software and statistics*

I suggest the following modification to the 2 ^nd^ sentence.

“was used for preparing graphs and preforming statistical calculations”.

Author´s response: It has been corrected.

*Ethics approval*

no suggestions

**RESULTS** 

*IPNV did not infect trout RBCs*

I suggest the following recasting of the last sentence and addition of a new last sentence.

“Moreover, flow cytometry analysis of control and IPNV-exposed RBCs for IPNV VP3 protein did not show significant differences (Figure 1C and D).  Therefore, IPNV did not infect trout RBCs.”

Author´s response: We have recasted the sentence as advised.

*IPNV exposure increased the expression of interferon-related antiviral immune genes and proteins in RBCs*

The first sentence should be shortened as follows

“to determine if IPNV would induce an antiviral response in RBCs.”

Are there antiviral genes that are not immune genes?

In other words, can the description be simplified to just ‘antivrial genes”?

Author´s response: We have shortened the sentence and the description simplified to antiviral genes

**DISCUSSION**

*2* *^nd^* *paragraph*

The last two sentences could refer to the data of the ms for support.

Author´s response: We have referred this affirmation to Figure 1.

*4* *^th^* *paragraph*

I wonder if the authors could briefly discuss the apparent constitutive expression of Mx in RBCs as seen in Panel D of Figure 2.  Also could Mx be released from RBCs?  In cattle Mx1 is found in exosomes (see Racicot K et al., 2012, Am J Reprod Immunol) and exosomes can be released by RBCs (see Danesh A et al., 2014, Blood).

Author´s response: Yes, Figure S1 also highlights the elevated constitutive expression of Mx in RBCs, compared to other CHSE-214.

Mx in RBCs exosomes is very interesting appreciation. However, Racicot et al (2012) concluded that “The results presented here show MX1 could play a role in the formation of vesicles secreted into the uterus and suggest involvement in basic cellular processes independent of its role during viral infections”. Therefore, although it is an interesting topic of investigation we would rather not to mention the possible link between Mx and RBCs exosomes in this manuscript since it is unknown whether it could be related to Mx antiviral function.

**FIGURES AND LEGENDS**

Figure 1

In panel A write out hpe on the X axis.

Author´s response: It has been written

Figure 2

Is the staining in the control of interest or just background?  I mentioned this earlier under the Discussion.  Could any of the cells in panel D be thrombocytes?

Author´s response: Yes, we have discussed about it in the discussion section. In relation to panel D, none of those cells are thrombocytes.

Figure 3

Could FFU be written out here in the legend so figure stands alone?  The legend mentions doing a two-way ANOVA but no mention of a post test is given, although the 1/5 supernatant dilution is identified with an asterisk?

Author´s response: Yes, FFU/mL has been deleted from panel A, x axis, as it is indicated in the figure legend. On the other hand, Sidak´s multiple comparison test was performed and has been added to the legend.

Figure 4  Title legend has a spelling mistake and is a bit misleading because panel B is not covered by the current title.  I suggest the following title.

“Reduction in RBC cytokine levels by IPNV and a possible mechanism”

The legend should give more information so the figure can better stand by itself.

Write out MFI (Mean Fluorescence Intensity) and FC (Flow Cytometry).

At two places in the legend, mention is made of the Mann-Whitney test but the outcome is not clearly stated.

Author´s response: Title of Figure 4 has been changed as follows: IPNV-exposure decreased cytokine levels in rainbow trout RBCs, and MFI and FC explained. In relation to the statistical analysis, Mann-Whitney test was performed for Fig4A and 4b, however, statistically significant differences were not observed.

# Peer review: Referee response for version 1

**DOI:** 10.5256/f1000research.14090.r27699

**Published:** 2017-11-20

**Authors:** Espen Rimstad, Maria Dahle

**Affiliations:** 1 Department of Food Safety and Infection Biology, Norwegian University of Life Sciences, Oslo, Norway; 2 Norwegian Veterinary Institute, Oslo, Norway

This is an interesting paper showing that purified rainbow trout RBC exposed to IPNV do not get infected by the virus, but nevertheless raise an innate antiviral response. The latter is shown by the induction of IFN and a few IFN related genes and that conditioned media from IPNV exposed RBC inhibits IPNV infection of the susceptibe cell line CHSE-214. Many methods are used to approach the hypotheses and the results appear provide new information on the very intriguing role of fish red blood cells in interaction with viruses, in particular by showing that the cells can induce an antiviral immune response without being infected.

 The main comments are related to how the authors have interpreted the results far beyond what they have shown, by drawing links to adaptive immune mechanisms and vaccination approaches, while they could have discussed antiviral protection mechanisms in further detail and with more scientific basis. There are also some missing information regarding the experimental details, and far too extensive use of an unpublished paper in the discussion of the data.   

 Comments:

1. The title is generalizing beyond the scope of the paper, and should specify the virus and the host studied (IPNV and onchorhynchus mykiss).
2. Abstract and Discussion: The statement that antiviral responses in RBCs could be targets for DNA vaccines is not linked to the data presented here. A vaccination effect requires antigen presentation and adaptive immune activation for long term protection, which is not touched upon at all in this paper. If this point should be made, the authors should in the discussion provide some explanation and scientific basis for  how they think  antiviral responses in RBCs to an uninfecting virus make them promising DNA vaccination targets, and how directing DNA vaccines to RBC should be  performed.
3. Introduction/Discussion: An unpublished paper: Nombela I, Puente-Marin S et al. is referred to in introduction and several times in the discussion. The content of this paper cannot be evaluated. The authors should primarily use published work in their discussion.
4. Introduction. There are many language mistakes in the introduction (and elsewhere). Please have the manuscript corrected linguistically. Trout is not a specific annotation.
5. Please specify that the work is performed for rainbow trout. Diseases are not agents. Diseases cause losses not loses. Viral titer not titters. *Aquabirnavirus* genus not *Aquabirnaviridae *genera, it is wrong suffix and it is not plural. etc.
6. The role of IPNV - VP3 is not completely unknown, there are several papers describing its function.
7. *“The VP2 epitopes are the target for antibodies against different serotypes.” *Not *against* but distinguishing between. It should be noted that the serotyping system is based upon the use of rabbit antisera, it is not based on the immune response from fish.
8. No observations of viral factories has been observed for ISAV.
9. *“The fact that these cells are nucleated* *implies that fish RBCs can respond to different stimulus and control cellular processes”. *Not really, having a nucleus doesn’t really prove that the cell can react various stimuli, that have to shown.
10. Materials and Methods: Number of fish used not given. Unclear if the fish used were tested for previous viral infections.
11. 18S is given as the reference gene target in the text, but in the table EF1a primers are given. Results is not clear on what was used.
12. Results: Unclear if the “n” in experiments is number of fish or parallel ex vivo infections.
13. *“IPNV segment A (IPNV-A) RNA levels inside RBCs and CHSE-214 cell line were similar at 1 and 3 hours post-exposure (hpe) (Figure 1A). After 3 hpe, IPNV-A RNA level was 4 logarithms lower in RBCs in comparison with CHSE-214 cells.” * Do you mean from 6 hpe and not after 3 hpe?  It is not 4 logs difference according to Fig 1 it is 3 log at 6 hpe and 5 log at 26 hpe. It is nevenr 4 logs?
14. What is the detection limit for VP3 in flow cytometri?
15. Discussion:
  1. The small trend towards a difference in cytokine levels Fig 4A should not be discussed as “downregulation”, and cannot be interpreted as a cell shut-off of the proteome by the infection. Most likely the slight trend towards down-regulation is just an effect of the relative upregulation of the IFN-I pathway (ISGs)
  2. Authors say in the discussion regarding the protective effect of conditioned media: “ *To elucidate which factors could be implicated in this protection, we evaluated the production of cytokines in IPNV-exposed RBCs*” It is well known that IFN-I, and not to the same degree cytokines, confers a direct antiviral response on surrounding cells. The upregulation of IFN-I is already shown at the transcriptional level, and the upregulation of ISGs directly indicates IFN activity. The main role of cytokines is to attract and activate immune cells and mobilize an immune response in tissue in vivo. The authors should clarify this to show that they understand the difference between local antiviral protection and crosstalk with the immune system. 

We have read this submission. We believe that we have an appropriate level of expertise to confirm that it is of an acceptable scientific standard, however we have significant reservations, as outlined above.

# Response

**Published:** 2017-12-01

**Authors:** Maria del Mar Ortega-Villaizan Romo

**Affiliations:** Universidad Miguel Hernandez, Spain

Dear Dr. Espen Rimstad and Dr. Maria Dahle, 

We appreciate very much your revision and constructive comments on the manuscript. We have included your corrections in the new version of the manuscript hoping that now the manuscript will be suitable for publication. 

Please find below the response to your comments:

1.    The title is generalizing beyond the scope of the paper, and should specify the virus and the host studied (IPNV and onchorhynchus mykiss).

Author’s response: The title have been changed as advised.

2.    Abstract and Discussion: The statement that antiviral responses in RBCs could be targets for DNA vaccines is not linked to the data presented here. A vaccination effect requires antigen presentation and adaptive immune activation for long term protection, which is not touched upon at all in this paper. If this point should be made, the authors should in the discussion provide some explanation and scientific basis for  how they think  antiviral responses in RBCs to an uninfecting virus make them promising DNA vaccination targets, and how directing DNA vaccines to RBC should be  performed.

Author’s response: In order to avoid misunderstanding we have deleted DNA vaccines from abstract

3.    Introduction/Discussion: An unpublished paper: Nombela I, Puente-Marin S et al. is referred to in introduction and several times in the discussion. The content of this paper cannot be evaluated. The authors should primarily use published work in their discussion.

Author’s response: The paper reference can be included now and has been included.

4.    Introduction. There are many language mistakes in the introduction (and elsewhere). Please have the manuscript corrected linguistically. Trout is not a specific annotation.

Author’s response: the manuscript has been linguistically corrected and trout has been properly annotated as rainbow trout.

5.    Please specify that the work is performed for rainbow trout. Diseases are not agents. Diseases cause losses not loses. Viral titer not titters. Aquabirnavirus genus not Aquabirnaviridae genera, it is wrong suffix and it is not plural. etc.

Author’s response: we have specified that the work has been done for rainbow trout. Titer has been written instead of titters. Aquabirnavirus genus has been written instead of Aquabirnaviridae genera.

6.    The role of IPNV - VP3 is not completely unknown, there are several papers describing its function.

Author’s response: This sentence has been deleted since it is not the target of our study.

7.    “The VP2 epitopes are the target for antibodies against different serotypes.” Not against but distinguishing between. It should be noted that the serotyping system is based upon the use of rabbit antisera, it is not based on the immune response from fish.

Author’s response: This sentence has been deleted since it is not the target of our study.

8.    No observations of viral factories has been observed for ISAV.

Author’s response: We have deleted that sentence.

9.    “The fact that these cells are nucleated implies that fish RBCs can respond to different stimulus and control cellular processes”. Not really, having a nucleus doesn’t really prove that the cell can react various stimuli, that have to shown.

Author’s response: We have deleted that sentence.

10.    Materials and Methods: Number of fish used not given. Unclear if the fish used were tested for previous viral infections.

Author’s response: The number of fish is indicated in each experiment/figure. On the other hand, the fish has not been tested for previous infection. However, the fish farm appears to be free of IPNV and VHSV, since they do not have associated mortalities.

11.    18S is given as the reference gene target in the text, but in the table EF1a primers are given. Results is not clear on what was used.

Author’s response: Yes, this has been an error. EF1a has been removed from the primers table.

12.    Results: Unclear if the “n” in experiments is number of fish or parallel ex vivo infections.

Author’s response: Yes, “n” refers to number of fish/ ex vivo infections

13.    “IPNV segment A (IPNV-A) RNA levels inside RBCs and CHSE-214 cell line were similar at 1 and 3 hours post-exposure (hpe) (Figure 1A). After 3 hpe, IPNV-A RNA level was 4 logarithms lower in RBCs in comparison with CHSE-214 cells.”  Do you mean from 6 hpe and not after 3 hpe?  It is not 4 logs difference according to Fig 1 it is 3 log at 6 hpe and 5 log at 26 hpe. It is nevenr 4 logs?

Author’s response: Yes, please excuse the error. We have corrected it as “After 6 hpe, IPNV-A RNA level was 3 logarithms lower”

14.    What is the detection limit for VP3 in flow cytometri?

Author’s response: We do not know the limit of detection for VP3 by flow cytometry. However, by ELISA, it has been described that the limit of the detection for 2F12 + 3B12 antibody mix (anti-VP3) is 104 TCID50/ml (Domínguez J, Hedrick RP, Sánchez-Vizcaino JM: Use of monoclonal-antibodies for detection of infectious pancreatic necrosis virus by the enzyme-linked-immunosorbent-assay (ELISA). Dis Aquat Organ. 1990; 8: 157–63. Publisher Full Text).

15.    Discussion:

1.    The small trend towards a difference in cytokine levels Fig 4A should not be discussed as “downregulation”, and cannot be interpreted as a cell shut-off of the proteome by the infection. Most likely the slight trend towards down-regulation is just an effect of the relative upregulation of the IFN-I pathway (ISGs)

Author’s response: We have included this appreciation in the discussion.

2.    Authors say in the discussion regarding the protective effect of conditioned media: “To elucidate which factors could be implicated in this protection, we evaluated the production of cytokines in IPNV-exposed RBCs” It is well known that IFN-I, and not to the same degree cytokines, confers a direct antiviral response on surrounding cells. The upregulation of IFN-I is already shown at the transcriptional level, and the upregulation of ISGs directly indicates IFN activity. The main role of cytokines is to attract and activate immune cells and mobilize an immune response in tissue in vivo. The authors should clarify this to show that they understand the difference between local antiviral protection and crosstalk with the immune system. 

Author’s response: In order to avoid misunderstandings, and due to lack of information about il8, il1b and tnfa receptor pathways in CHSE-214, we have deleted in the manuscript the relation between il8, il1b and tnfa with the protection of CHSE against IPNV.
